# Association between female waist-hip ratio and live birth in patients undergoing *in vitro* fertilization: a retrospective cohort study

**DOI:** 10.3389/fendo.2025.1537360

**Published:** 2025-02-27

**Authors:** Mingming Ye, Yingying Yang, Chenting Cai, Zhen Li, Andong Qiu, Jia He, Jing Ma, Orhan Bukulmez, Robert J. Norman, Xiaoming Teng, Miaoxin Chen

**Affiliations:** ^1^ Centre for Assisted Reproduction, Shanghai Key Laboratory of Maternal Fetal Medicine, Shanghai Institute of Maternal-Fetal Medicine and Gynecologic Oncology, Shanghai First Maternity and Infant Hospital, School of Medicine, Tongji University, Shanghai, China; ^2^ Clinical Research Unit, Shanghai Key Laboratory of Maternal Fetal Medicine, Shanghai Institute of Maternal-Fetal Medicine and Gynecologic Oncology, Shanghai First Maternity and Infant Hospital, School of Medicine, Tongji University, Shanghai, China; ^3^ School of Medicine, Tongji University, Shanghai, China; ^4^ Department of Endocrinology and Metabolism, Renji Hospital, Shanghai Jiao Tong University School of Medicine, Shanghai, China; ^5^ Division of Reproductive Endocrinology and Infertility, Department of Obstetrics and Gynecology, The University of Texas Southwestern Medical Center, Dallas, TX, United States; ^6^ Robinson Research Institute, School of Pediatrics and Reproductive Health, The University of Adelaide, Adelaide, SA, Australia

**Keywords:** *In vitro* fertilisation, central obesity, waist-hip ratio, body mass index, live birth rate

## Abstract

**Background:**

Maternal obesity is associated with adverse pregnancy outcomes. It negatively affects IVF/ICSI outcomes and offspring health. However, it is unclear whether waist-hip ratio (WHR) has an impact on outcomes of *in vitro* fertilization (IVF) or intracytoplasmic sperm injection (ICSI) cycles.

**Methods:**

A retrospective cohort study screened 943 patients who underwent IVF/ICSI treatment between February and June 2020 in Shanghai, China, and 828 patients were finally included in the analyses. The body weight, height, waist circumference and hip circumference were measured before ovarian stimulation, and their IVF/ICSI outcomes were followed up. The cut-off point of WHR was determined by the area under the receiver operating characteristic (ROC) curve. Live birth rate from the first embryo transfer cycle was the primary outcome. The secondary outcomes included cumulative live birth, miscarriage rate and birthweight.

**Results:**

Women with relatively high WHR (≥0.783) showed lower live birth rate (adjusted odds ratio (aOR): 0.657, 95%CI: 0.466-0.926), lower cumulative live birth rate (aOR: 0.580, 95%CI: 0.413-0.814), and higher miscarriage rate (aOR=2.865, 95%CI: 1.300-6.316) as compared with those with low WHR (<0.783), independently of BMI. Joint WHR and BMI analyses showed that, compared with the reference group (those with low WHR and normal weight), those with high WHR and normal BMI had lower live birth rate (aOR=0.653, 95%CI: 0.447-0.954) and cumulative live birth rate (aOR=0.600, 95%CI: 0.413-0.872), and higher miscarriage rate (aOR=2.865, 95%CI: 1.229-6.676), Whereas the patients with both high WHR and high BMI only showed a significant lower cumulative live birth rate (aOR=0.612, 95%CI: 0.404-0.926). Moreover, there was no significant association between BMI and pregnancy outcomes, or between maternal WHR and birth weights.

**Conclusions:**

Our results demonstrated that higher WHR was associated with lower fecundability in women undergoing IVF/ICSI cycles, independently of BMI. Interestingly, the adverse effects of central obesity were more evident in patients with lower BMI. Thus WHR appears to be a better predictor of female fertility treatment outcomes as compared with BMI.

## 1 Introduction

Worldwide, overweight and obesity are growing at an alarming rate and has nearly doubled since 1975, accounting for a third of the world’s population (1, 2). Overweight and obesity is typically classified by body mass index (BMI) and associated with an increased risk of non-communicable diseases and premature death (3). A retrospective observational study showed a 50% decline of delivery rate in women with BMI>40kg/m^2^ who underwent *in-vitro* fertilization (IVF) (4). Compared with the normal weight peers, obese women have significantly lower live birth rate following IVF (5). However, some studies did not observe the negative association between increased BMI and IVF outcomes (6–8).

The relationship between BMI and body fat content varies among different ethnic groups. As compared with Caucasians of the same sex, age and BMI, Asian people generally have higher proportion of body fat (9), which will result in an underestimate of health risks. Usually, body fat content and fat distribution are considered important indicators of health risk. Dual-energy x-ray absorptiometry (DEXA) is recognized as the gold standard for quantifying abdominal fat mass (10). However, the complexity and high cost of DEXA extremely limits its routine clinical use. Recently, waist circumference (WC) or waist hip ratio (WHR), which could be more easily measured, is recommended as an index for central obesity by the World Health Organization (WHO) (11). Interestingly, compared with BMI, WHR is more closely associated with metabolic risk factors, cardiovascular morbidity and mortality (12).

WHR was positively associated with the risk of female infertility (13). To date, few studies have examined the effect of WHR on IVF outcomes. A prospective cohort study involving 542 women in the Netherlands showed that increased WHR was associated with the decreased odds of conception per artificial insemination cycle (14). However, a study of Asian women (Chinese, Malay and Indian) showed that central obesity indices (including WHR) were not associated with fecundability (15). Thus, it is still unclear whether WHR is associated with IVF outcomes.

This study aimed to investigate the association between WHR and pregnancy outcome including live birth, cumulative live birth, miscarriage and neonatal outcomes in women undergoing IVF/intracytoplasmic sperm injection (ICSI) cycles.

## 2 Materials and methods

### 2.1 Participants

**Figure 1:**
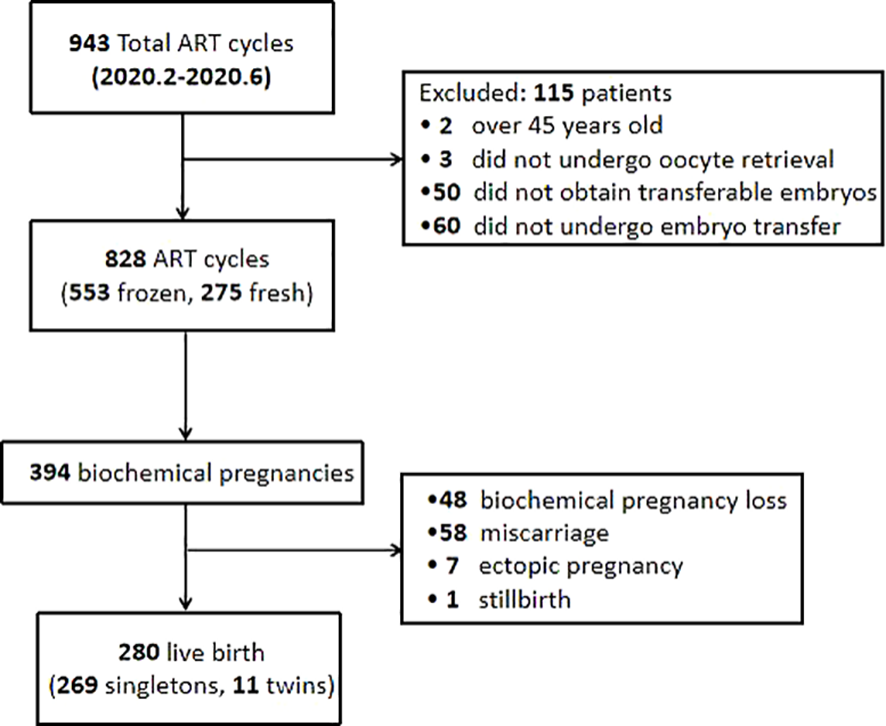
Flowchart of patients included in this study.

This retrospective study screened 943 patients who underwent IVF or ICSI treatment between February and June of 2020 in the Centre for Assisted Reproduction of Shanghai First Maternity and Infant Hospital. Patients were excluded for the study if they: (1) were over 45 years old (N=2), (2) did not undergo oocyte retrieval (N=3), (3) did not obtain transferable embryos (N=50), or (4) did not undergo embryo transfer (N=60). The flowchart of participants included in this study is shown in **Figure 1**. This study was approved by the Research Ethics Committee of Shanghai First Maternity and Infant Hospital (KS21211).

### 2.2 IVF procedures

All included patients had received controlled ovarian stimulation (COS), including long GnRH agonist (GnRH-a) protocol, GnRH antagonist (GnRH-ant) protocol, short GnRH-a protocol, mild stimulation protocol and progestin-primed ovarian stimulation (PPOS) protocol. The details of each protocol were described previously (16). Oocytes were retrieved 34-36 h after HCG injection. The process of semen preparation, conventional IVF/ICSI, embryo culture and assessment were performed as described previously (17). For women who underwent fresh embryo transfer, one or two high-quality embryos were transferred three or five days after oocyte retrieval under transabdominal ultrasound guidance. Luteal phase support was started on the day of oocyte retrieval as described previously (16). Endometrial preparation protocols and luteal phase support for frozen-thawed embryo transfer were described previously (16). Luteal-phase support was maintained in women with a positive hCG test until ten weeks of pregnancy.

### 2.3 Anthropometric measurements

All subjects were measured in light clothes without shoes and hats. Body weight was measured by weight scale to the nearest 0.1kg. Height was measured in meters by a rangefinder. A tapeline was used to measure the WC and hip circumference to the nearest 0.1cm. The WC was measured at the narrowest part of the torso, which is mid-way between the inferior border of the rib cage and the superior aspect of the iliac crest. The hip circumference was measured at the level of the trochanters, which is the maximal extension of the buttocks. WHR was calculated by dividing waist circumstance by hip circumference. Weight, height, waist and hip circumference were measured twice by a nurse throughout the study, and the mean value of these measurements was used in our study to minimize errors.

### 2.4 Outcomes and covariates

The primary outcome was live birth after the first embryo transfer of the IVF cycles, defined as the delivery of one or more living infants (≥22 weeks gestation or birth weight more than 500 g). Cumulative live birth refers to the sum of deliveries with at least one live birth in the current IVF cycle including all fresh and/or frozen embryo transfers until one live birth was delivered or until all embryos were used, whichever occurs first (18). Biochemical pregnancy was defined as serum beta-hCG≥10 IU/L 14 days after embryo transfer. Biochemical pregnancy loss was defined as a pregnancy diagnosed only by the detection of beta-hCG in serum without any ultrasonographical evidence. Clinical pregnancy was defined when at least one gestational sac was observed on ultrasonography 7 weeks after embryo transfer with or without heart beat, including ectopic pregnancy. Multiple pregnancies were defined as a pregnancy with two or more gestational sacs or positive heart beats 7 weeks after embryo transfer. Miscarriage was defined as the spontaneous loss of an intra-uterine pregnancy prior to 22 weeks gestation. Stillbirth was defined as fetal death occurring during late pregnancy (at 22 completed weeks of gestational age and later) or during childbirth. Preterm birth (PTB) was defined as births that took place before 37 weeks gestation. Low birth weight (LBW) and macrosomia were identified as birthweight <2500g and >4000g, respectively. Small for gestational age (SGA) was identified as birthweight <10th percentile and large for gestational age (LGA) was identified as birthweight >90th percentile, based on Chinese reference singleton newborns stratified by gestational age and sex at birth (19).

The cut-off point of BMI ≥24kg/m^2^ and BMI ≥28kg/m^2^ were adopted for overweight and obesity respectively as recommended for the Chinese population (20). Other variables which may affect the outcomes of IVF/ICSI were also included in the analysis, including female age, educational level, type of infertility, factors of infertility, number of previous IVF cycles, duration of infertility, stimulation protocol, duration of stimulation, total gonadotrophin dose, number of oocytes retrieved, fertilization method, type of embryo transfer, numbers of embryos transferred, and endometrial thickness on embryo transfer day.

### 2.5 Statistical analyses

Demographic characteristics of the participants were described as means ± standard deviations (SD) for continuous variable, when they met normal distribution, otherwise, median and interquartile ranges were reported. Counts (percentages) were presented for categorical variables. Students’ t-test was applied to compare the difference between two groups for normally distributed variables. Chi-squared tests were used to compare qualitative data, and Fisher’s exact test was applied when the expected frequency was less than five. The dataset included in this study had no missing data. Data management and statistical analysis was performed using SAS statistical analysis software, version 9.4 (SAS Institute, Inc). Two-sided *p*<0.05 were considered as statistically significant.

The optimal cut point for female WHR was determined by evaluating the area under the receiver operating characteristic (ROC) curve in univariable logistic regression model with a live birth event as a dependent variable and the female WHR (continuous variable) as an independent predictor. The optimal cut point (0.783) was selected, which has the highest Youden Index. Then the female WHR was categorized into low (0.62-<0.783) and high (0.783-<1.09). Univariable logistic regressions and multiple logistic regression models were performed to determine the impacts of female WHR on pregnancy outcomes including live birth, cumulative live birth and miscarriage. The multiple logistic regression models for live birth and miscarriage were adjusted for female age, female education level, female BMI, type of embryo transfer, endometrial thickness, type of infertility, factors of infertility, number of IVF cycle, stimulation protocol, fertilization method, stage of embryo transferred, number of embryo(s) transferred, male WHR. The multiple logistic regression models for cumulative live birth were adjusted for female age, female education level, female BMI, type of infertility, factors of infertility, number of IVF cycle, stimulation protocol, fertilization method, and male WHR. The relationship between female BMI and pregnancy outcomes was analyzed in the same way.

We categorized female WHR as low and high, and weight as normal (BMI: 18.5-<24kg/m^2^) and overweight (BMI ≥24kg/m^2^) separately. Then we created a 4-category variable to represent the joint WHR and BMI. In the analysis of joint WHR and BMI with live birth, cumulative live birth and miscarriage, participants with low WHR and normal weight were set as the reference group, and similar covariates were adjusted in the logistic regression models. The area under the curve (AUC) of the female waist-hip ratio with that of BMI was performed by univariable regression models. The interaction between female BMI and waist-hip ratio was analyzed according to the multivariate regression.

In order to confirm the effects of central obesity on IVF/ICSI outcomes, WC was also analyzed in the same way with WHR, while the optimal cut-off value of waist circumference was 74.5cm. Then the female WC was categorized into low (56.0-<74.5cm) and high (74.5-<110.0cm).

## 3 Results

In total, 828 couples were included in the study. Since we didn’t observe significant associations between male factors and pregnancy outcomes, only the associations between female characteristics were presented. The mean age of the females was 32.4 ± 4.2 years. The average BMI and WHR of women were 22.8 ± 3.2 kg/m^2^ and 0.82 ± 0.06, respectively. The percentage of overweight and obesity was 22.5% and 7.5% in this cohort. Moreover, women with higher WHR tended to use higher doses of medication during stimulation. In addition, WHR was inversely associated with the number of oocytes collected. The characteristics of the participants are shown in **Table 1**.

**Table 1:** Demographic characteristics of the patients.

	Whole cohort (N=828)	Female waist-hip-ratio		P-value
		Low (N=239)	High (N=589)	
Female age (years)	32.4 ± 4.2	32.1 ± 3.9	32.6 ± 4.3	0.100
Female education level				0.011
≤High school	236 (28.5)	51 (21.3)	185 (31.5)	
Junior College degree	185 (22.4)	51 (21.3)	134 (22.8)	
College degree	304 (36.8)	101 (42.3)	203 (34.5)	
Postgraduate degree	102 (12.3)	36 (15.1)	66 (11.2)	
Body mass index (kg/m^2^)	22.8 ± 3.2	21.0 ± 2.2	23.5 ± 3.2	<0.001
<18.5	36 (4.3)	25 (10.5)	11 (1.9)	<0.001
18.5-<24	544 (65.7)	192 (80.3)	352 (59.8)	
24-<28	186 (22.5)	19 (8.0)	167 (28.4)	
≥28	62 (7.5)	3 (1.3)	59 (10.0)	
Female waist-hip-ratio	0.82 ± 0.06	0.75 ± 0.02	0.85 ± 0.05	<0.001
Male Waist-hip-ratio	0.90 ± 0.06	0.89 ± 0.06	0.90 ± 0.06	0.002
Duration of infertility (years)	2 (1-4)	2 (1-4)	2 (1-4)	0.392
Type of infertility (%)				0.249
Primary	436 (52.7)	134 (56.1)	302 (51.4)	
Secondary	391 (47.3)	105 (43.9)	286 (48.6)	
Factors of infertility (%)				0.664
Male factor	225 (27.2)	59 (24.7)	166 (28.2)	
Female factor	522 (63.1)	157 (65.7)	365 (62.0)	
Combined factor	66 (8.0)	20 (8.4)	46 (7.8)	
Unexplained	14 (1.7)	3 (1.3)	11 (1.9)	
Number of IVF cycle (%)				0.878
First	619 (74.8)	176 (73.6)	443 (75.2)	
Second	114 (13.8)	35 (14.6)	79 (13.4)	
≥Third	95 (11.5)	28 (11.7)	67 (11.4)	
Stimulation protocol (%)				0.565
GnRH agonist	234 (28.3)	69 (28.9)	165 (28.0)	
GnRH antagonist	317 (38.3)	85 (35.6)	232 (39.4)	
Other protocols	277 (33.4)	85 (35.6)	192 (32.6)	
Duration of stimulation (days)	9 (8-11)	9 (8-11)	9 (8-12)	0.003
Starting gonadotropin dose (IU)	225 (150-225)	187 (150-225)	225 (150-225)	0.393
Total gonadotropin dose (IU)	1800 (1500-2300)	1800 (1350-2175)	1875 (1500-2475)	<0.001
Number of oocytes retrieved	9 (5-14)	10 (7-15)	9 (5-14)	0.001
Fertilisation method (%)				0.047
IVF	450 (54.3)	117 (49.0)	333 (56.5)	
ICSI	378 (45.7)	122 (51.0)	256 (43.5)	
Fertilisation rate	0.80 (0.64-1.00)	0.78 (0.65-0.92)	0.80 (0.63-1.00)	0.705
Cleavage rate	1 (1-1)	1 (1-1)	1 (1-1)	0.251
Available embryo rate	0.50 (0.38-0.71)	0.50 (0.36-0.71)	0.50 (0.38-0.71)	0.649
Type of embryo transfer				0.127
Frozen	553 (66.8)	169 (70.7)	384 (65.2)	
Fresh	275 (33.2)	70 (29.3)	205 (34.8)	
Stage of embryos transferred				0.046
Cleavage	608 (73.4)	164 (68.6)	444 (75.4)	
Blastocyst	220 (26.6)	75 (31.4)	145 (24.6)	
Number of embryos transferred				0.991
One	745 (90.0)	215 (90.0)	530 (90.0)	
Two	83 (10.0)	24 (10.0)	59 (10.0)	
Endometrial thickness (mm)	10.0 (9.0-12.0)	10.0 (8.7-12.0)	10.0 (9.0-12.0)	0.960
<8	59 (7.1)	20 (8.4)	39 (6.6)	0.376
≥8	769 (92.9)	219 (91.6)	550 (93.4)	

The pregnancy outcomes of patients are shown in **Table 2**. The relationship between BMI and pregnancy outcomes was not presented as there was no statistical difference (**Supplementary Table S1**). The live birth rate was 33.8% and the miscarriage rate was 7.0% in the whole cohort. Compared with women with relatively high WHR (≥0.783), those with low WHR (<0.783) showed higher rates of live birth (40.2% *vs*. 31.2%, P=0.014) and cumulative live birth (59.0% *vs*. 47.0%, P=0.002), and lower rates of miscarriage (3.4% *vs*. 8.5%, P=0.009). We did not observe statistical significance in other outcomes between the two groups such as biochemical pregnancy rate and clinical pregnancy rate (P > 0.05, **Table 2**).

**Table 2:** Pregnancy outcomes of patients in different waist-hip ratio groups.

	Whole cohort (N=828)	Female waist-hip ratio		P-value
		Low (N=239)	High (N=589)	
Biochemical pregnancy	394 (47.6)	119 (49.8)	275 (46.7)	0.418
Biochemical pregnancy loss	48 (5.8)	14 (5.9)	34 (5.8)	0.962
Clinical pregnancy	346 (41.8)	105 (43.9)	241 (40.9)	0.425
Multiple pregnancy	21 (2.5)	5 (2.1)	16 (2.7)	0.605
Miscarriage	58 (7.0)	8 (3.4)	50 (8.5)	0.009
Ectopic pregnancy	7 (0.9)	1 (0.4)	6 (1.0)	0.680
Stillbirth	1 (0.1)	0	1 (0.2)	1.000
Live birth	280 (33.8)	96 (40.2)	184 (31.2)	0.014
Twins	11 (1.3)	3 (1.3)	8 (1.4)	1.000
Cumulative live birth	418 (50.5)	141 (59.0)	277 (47.0)	0.002

Univariate regression and multivariate regression analyses showed similar results (**Table 3**). After adjusting for female age, female BMI and other potential confounders, the multivariable logistic regression analyses showed that female WHR was negatively associated with live birth rate and cumulative live birth rate with the adjusted odds ratio (aOR) at 0.657 (95%CI: 0.466-0.926) and 0.580 (95%CI: 0.413-0.814) respectively, and positively associated with miscarriage rate (aOR=2.865, 95%CI: 1.300-6.316) (**Table 3**).

**Table 3:** Multivariable analysis for live birth, miscarriage and cumulative live birth.

	Univariable regression				Multiple regression#			
	cOR	95% CI		P value	aOR	95% CI		P value
Live birth								
Female Waist-hip ratio (High vs Low)	0.677	0.495	0.924	0.014	0.657	0.466	0.926	0.016
Miscarriage								
Female Waist-hip ratio (High vs Low)	2.678	1.250	5.738	0.011	2.865	1.300	6.316	0.009
Cumulative Live birth								
Female Waist-hip ratio (High vs Low)	0.617	0.455	0.837	0.002	0.580	0.413	0.814	0.002

cOR, crude odds ratio; 95%CI, 95% confidence interval; aOR, adjusted odds ratio.

#Regression Model for live birth and miscarriage was adjusted for female age, Female education level, female BMI, Type of embryo transfer, endometrial thickness, type of infertility, factors of infertility, number of IVF cycle, stimulation protocol, fertilization method, stage of embryo transferred, number of embryo(s) transferred, male Waist-hip ratio.

Regression Model for cumulative live birth was adjusted for female age, Female education level, female BMI, type of infertility, factors of infertility, number of IVF cycle, stimulation protocol, fertilization method, total number of embryo(s) transferred, male Waist-hip ratio.

The associations of joint WHR and BMI with live birth, cumulative live birth and miscarriage were shown in **Table 4**. There was no significant difference between the reference group with low WHR and normal weight and the group with low WHR and overweight. Compared with the reference group, the group with high WHR and normal weight had lower live birth rate (aOR=0.653, 95%CI: 0.447-0.954) and cumulative live birth rate (aOR=0.600, 95%CI: 0.413-0.872) and higher miscarriage rate (aOR=2.865, 95%CI: 1.229-6.676). In contrast, participants with both high WHR and BMI only showed a significant lower cumulative live birth rate (P=0.020), and a trend towards lower live birth rate (P=0.078) as well as a higher miscarriage rate (P=0.111). Besides, waist-hip ratio (high *vs* low) showed the greater value of AUC than BMI (**Table 5**). There was no interaction between female BMI and waist-hip ratio according to the multivariate analyses for live birth, miscarriage as well as cumulative live birth (**Supplementary Table S2**). The P value of Hosmer and Lemeshow goodness of fit test for the multivariable logistic regression was 0.959, suggesting the model had high goodness of fit.

**Table 4:** Odds ratios of live birth, miscarriage and cumulative live birth based on the joint multivariable regression analysis.

Female Waist-hip ratio	Female BMI	N	Live birth				Miscarriage				Cumulative Live birth			
			aOR	95% CI		P value	aOR	95% CI		P value	aOR	95% CI		P value
Low	Normal weight (18.5-<24)	192	ref	–	–	–	Ref	–	–	–	ref	–	–	–
	Overweight (≥24)	22	1.321	0.525	3.323	0.555	1.326	0.153	11.528	0.798	1.823	0.668	4.976	0.241
High	Normal weight (18.5-<24)	352	0.653	0.447	0.954	0.027	2.865	1.229	6.676	0.015	0.600	0.413	0.872	0.007
	Overweight (≥24)	226	0.683	0.448	1.043	0.078	2.131	0.841	5.400	0.111	0.612	0.404	0.926	0.020

aOR, adjusted odds ratio; 95%CI, 95% confidence interval.

#Regression Model for live birth and miscarriage was adjusted for female age, Female education level, Type of embryo transfer, endometrial thickness, type of infertility, factors of infertility, number of IVF cycle, stimulation protocol, fertilization method, stage of embryo transferred, number of embryo(s) transferred, male Waist-hip ratio.

Regression Model for cumulative live birth was adjusted for female age, Female education level, type of infertility, factors of infertility, number of IVF cycle, stimulation protocol, fertilization method, total number of embryo(s) transferred, male Waist-hip ratio.

**Table 5:** AUC of the female waist-hip ratio and BMI with pregnancy outcomes from univariable regression.

	Live birth	Miscarriage	Cumulative Live birth
Waist-hip ratio (High vs Low)	0.541	0.581	0.549
BMI (overweight vs normal weight)	0.510	0.508	0.517

Moreover, we separately analyzed the relationship between female waist circumference and IVF/ICSI outcomes (**Supplementary Tables S3**–**S5**). Consistent with the WHR analysis, higher waist circumference was associated with lower live birth rate (aOR=0.635, 95%CI: 0.445-0.907) and cumulative live birth rate (aOR=0.592, 95%CI: 0.422-0.831) (**Supplementary Table S4**). Women with normal BMI and higher waist circumference also had significant lower live birth (aOR=0.636, 95%CI: 0.437-0.924) and cumulative live birth (aOR=0.642, 95%CI: 0.451-0.915) rates than those with normal BMI and low waist circumference (**Supplementary Table S5**). In addition, we did not observe any associations between BMI (neither continuable variable nor BMI categorized using Chinese/Asian/WHO criteria) and live birth in the current population (9, 20).

To further investigate the effect of maternal WHR on offspring, we included neonatal outcomes in our analysis. Although the offspring of women with high WHR had higher mean birthweight than those with low WHR, the difference between the groups was not statistically significant (**Supplementary Table S6**).

## 4 Discussion

### 4.1 Main findings

This study showed that Chinese women with WHR values above 0.783 had lower live birth rate with a higher miscarriage rate and lower cumulative live birth rate compared with women having WHR less than 0.783, independently of BMI. However, there was no significant associations between BMI and IVF pregnancy outcomes. These results suggest that WHR may be a better predictor of adverse outcomes than BMI in women who underwent IVF/ICSI treatments.

### 4.2 Interpretation

Evidence suggests that obesity has a negative impact on both the follicle environment and embryo development, which may contribute to impaired endometrial decidualization and placental abnormalities (21–23). The levels of free fatty acids, markers of inflammation, and insulin resistance are increased in the follicular environment of women with obesity who underwent IVF (24). In addition, central obesity disrupts the stability of the hypothalamic-pituitary-ovarian axis by affecting the secretion of leptin, reactive oxygen species and other adipokines, which further exacerbates metabolic and reproductive abnormalities (25). Moreover, abdominal obesity may lead to an imbalance in the secretion of sex hormones, especially androgens, which is known to cause infertility in women (26).

As the most widely used indicator of obesity, BMI is closely related to female fertility (5, 27). However, consistent with previous studies (6–8), there were no significant associations between elevated BMI and IVF outcomes in our study. For example, the OR between BMI and cumulative live birth were not statistically significant, whether WHR was adjusted in the multivariate regression analysis model or not. These results may be due to the differences in the study population, BMI values, sample size, selection bias or analysis methods. For instance, only 30% of women in this study were overweight or obese and most were of normal weight.

When comparing women with similar BMI, those with central obesity had higher risk of anovulation (28). A retrospective study of Chinese women found that normal weight obesity (normal BMI but a high percentage of body fat) was associated with lower number of retrieved oocytes, fertilized oocytes, cleaved embryos and good-quality embryos (29). Women undergoing IVF with higher WC had lower live birth rate and higher spontaneous miscarriage rate (30, 31). In contrast, the reduction in WC was associated with an increased odds of pregnancy (32). A prospective cohort study observed delayed conception among black women in the United States with high WHR (≥0.85) after controlling for BMI (33). The pregnancy rate of IVF cycles was lower in women with WHR ≥0.80 than those with gynoid body shape, independent of BMI (34). However, the effects of WHR on major IVF outcomes, such as live birth are not clear. Our study showed that women with relatively high WHR (≥0.783) were associated with lower live birth rate and higher miscarriage rate, regardless of BMI. Since WHR was not associated with biochemical or clinical pregnancy rates, our data suggest that lower live birth rate was due to higher miscarriage rate in women with central obesity. Consistently, higher WC was associated with lower live birth rate after adjusting for BMI and other confounding factors in this study. We further compared the pregnancy outcome AUCs of the female waist-hip ratio with that of BMI. It turned out that the waist-hip ratio (high *vs* low) showed the highest AUCs. And there was no interaction between WHR and BMI regardless of pregnancy outcomes, which suggested that WHR appears to be a better predictor of female fertility treatment outcomes.

Interestingly, we found that the number of ampoules of gonadotrophin (both initial and total) administered was significantly higher in women with higher WHR, which had not been previously reported. Souter et al. had previously reported heavier women tended to need higher dose of gonadotropins during the stimulation protocol (35). And the mean number of oocytes collected was negatively related to female body weight (6), which is consistent with our findings.

Since even non-obese Asians show increased health risk associated with body fat percentage, common BMI indicators do not apply to Asians. We used the Chinese criteria of overweight (BMI ≥ 24 kg/m²) and obesity (BMI ≥ 28 kg/m²) in this study (20), which is higher than the WHO criteria for Asians. Previous studies have reported different thresholds for WHR threshold ranging from 0.80 to 0.85 in women (36). However, there is no consensus for WHR threshold in different ethnic populations. In our study, we observed a lower WHR threshold (≥0.783) in this cohort, indicating that Chinese women may have a lower WHR threshold as an index of central obesity. When taking both WHR and BMI into account, we found that women of normal weight but high WHR showed statistically significant reduction in fertility, which suggested that WHR rather than BMI was associated with adverse IVF outcomes for Chinese women.

With substantial improvements in cryopreservation and frozen embryo transfer techniques, cumulative live birth rate has been considered a better indicator of the quality and success of IVF by both the patients and the clinicians (37). As the yield of oocyte increases, the cumulative live birth rate increased significantly. Conversely, the reduction of oocyte quality was associated with lower cumulative live birth rate (38). Maternal obesity can lead to a decline in oocyte quality (39). However, the relationship between central obesity and cumulative live birth rate had never been reported before. Our findings indicated that women with higher WHR were associated with lower cumulative live birth rate.

Maternal overnutrition will change the environmental conditions *in utero*, leading to altered epigenetic modifications, which further affects offspring metabolism (40). Increasing evidence suggests that children of obese mothers have an increased risk of obesity, partly due to maternal overnutrition leading to an oversupply of fatty acids and glucose to the fetus (41, 42). Other studies have also reported that maternal overweight/obesity is associated with an increased risk of high birthweight and subsequent overweight/obesity and fatty liver in offspring (43, 44). For every 0.1 unit increase in maternal WHR, the birthweight of offspring increased by 120 g (45). However, the effects of maternal WHR on IVF offspring have not been reported in the literature. Although we observed some minor differences in the results, it was not statistically significant and this may be due to the low percentage of obesity in this study.

### 4.3 Strengths

This study has several strengths. To our knowledge, this is the first study mainly focused on the association between WHR and IVF/ICSI outcomes including live birth and cumulative live birth among Chinese women who underwent fresh or frozen embryo transfer. Different from the variable definition of “pregnancy rate”, live birth rate is the preferable standard for evaluating IVF outcomes. Besides, cumulative live birth rate is an important indicator that reflecting the status of all oocytes obtained from a single IVF/ICSI cycle, and the ultimate goal for patients. Notably, two thirds of women in this study were of normal weight, and our findings demonstrated that WHR is an independent factor that is associated with adverse IVF outcomes including the high risk of miscarriage and lower odds of live birth and cumulative live birth. Moreover, the anthropometric and covariate data were collected prior to ovarian stimulation, and most of patients (about 86%) subsequently underwent embryo transfer cycles. Additionally, to adjust for major confounders, multivariable logistic regression analysis was used to minimize bias.

### 4.4 Limitations

There are some limitations in this study. The main limitation is that the data came from a single IVF center as a retrospective study, which could only provide limited individual covariate information. Secondly, although WHR makes it easier to measure abdominal obesity than as compared to the complexity and high cost of DEXA, it is an anthropometric indicator and is almost constant for small weight fluctuations, and there may be measurement errors. Thirdly, for some patients who underwent frozen embryo transfers, there are variable time intervals between embryo transfer and oocyte retrieval. During this time, women’s WHR might have changed, thus might introduce error for the pregnancy outcomes estimation. Fourth, in clinical practice various protocols were utilized in the IVF/ICSI process, which might contribute to the potential biases in the study.

## 5 Conclusions

This retrospective cohort study showed that relatively high value of WHR (≥0.783) was associated with poorer IVF/ICSI outcomes, including increased risk of miscarriage, decreased rates of live birth and cumulative live birth, independent of BMI and other confounders. Joint WHR and BMI analyses suggest a strong association between central obesity and adverse IVF outcomes, highlighting that WHR may be of more concern than BMI when evaluating IVF/ICSI outcomes in the future. Although our study highlights the reproductive harms of central obesity in women, prospective studies are needed to validate our results. Furthermore, we need to determine a clinically significant WHR cut-off value, so as to provide better pre-conceptional counselling for women undergoing *in vitro* fertilization.

## Data Availability

The raw data supporting the conclusions of this article will be made available by the authors, without undue reservation.
