# Supplementary material for: Association between female waist-hip ratio and live birth in patients undergoing in vitro fertilization: a retrospective cohort study
Source: Front Endocrinol (Lausanne). 2025 Feb 27;16:1537360. doi: 10.3389/fendo.2025.1537360 (PMC11903291; doi:10.3389/fendo.2025.1537360)
Supplement: Supplementary file 1 [file Table1.doc]

**Supplemental Information**

**Association between female waist-hip ratio and live birth in patients undergoing in vitro fertilisation:** **A retrospective cohort study**

**Content:**

**Table S1.** The association between BMI and cumulative live birth from univariable and multiple regression with or without WHR

**Table S2.** Interaction analyses between female BMI and waist-hip ratio according to the multivariate regression

**Table S3.**Pregnancy outcomes of patients in different female Waist-Circumference groups.

**Table S4.** Multivariable analysis for live birth and cumulative live birth.

**Table S5.** Odds ratios of live birth and cumulative live birth based on the joint multivariable regression analysis.

**Table S6.** Neonatal outcomes of singletons in different waist-hip ratio groups.

**Table S1.** **The association between BMI and cumulative live birth from univariable and multiple regression with or without WHR**

|  | **Model without WHR**  **OR (95%CI)** | **P value** | **Model with WHR**  **OR (95%CI)** | **P value** |
| --- | --- | --- | --- | --- |
| **Univariable regression** | | | | |
| **BMI**  **(Over weight ≥24 vs**  **Normal weight 18.5-<24)** | **0.857 (0.635-1.158)** | **0.315** | **0.992 (0.724-1.357)** | **0.958** |
| **Waist-hip ratio**  **(high vs low)** | **-** | **-** | **0.572 (0.410-0.797)** | **0.001** |
| **Multiple variable regression** | | | | |
| **BMI**  **(Over weight ≥24 vs.**  **Normal weight 18.5-<24)** | **0.949 (0.687-1.299)** | **0.727** | **1.090 (0.783-1.518)** | **0.609** |
| **Waist-hip ratio (high vs low)** | **-** | **-** | **0.556 (0.392-0.788)** | **0.001** |

**Table S2. Interaction analyses between female BMI and waist-hip ratio according to the multivariate regression**

| **Parameter** | **Live birth** | | | | **Miscarriage** | | | | **Cumulative live birth** | | | |
| --- | --- | --- | --- | --- | --- | --- | --- | --- | --- | --- | --- | --- |
|  | **Estimate** | **Standard error** | **Wald chi-square** | **P value** | **Estimate** | **Standard error** | **Wald chi-square** | **P value** | **Estimate** | **Standard error** | **Wald chi-square** | **P value** |
| **Intercept** | **-2.03** | **0.63** | **10.49** | **0.001** | **-4.18** | **1.08** | **14.96** | **0.000** | **1.49** | **0.43** | **12.00** | **0.001** |
| **Waist-hip ratio (high)** | **-0.43** | **0.19** | **4.87** | **0.027** | **1.05** | **0.43** | **5.94** | **0.015** | **-0.51** | **0.19** | **7.18** | **0.007** |
| **BMI (over weight ≥24)** | **0.28** | **0.47** | **0.35** | **0.555** | **0.28** | **1.10** | **0.07** | **0.798** | **0.60** | **0.51** | **1.37** | **0.241** |
| **Waist-hip ratio (high) *BMI (over weight ≥24)** | **-0.23** | **0.51** | **0.21** | **0.646** | **-0.58** | **1.15** | **0.25** | **0.615** | **-0.58** | **0.54** | **1.15** | **0.285** |

**Table S3. Pregnancy outcomes of patients in different female Waist-Circumference groups**

|  | **Whole cohort (N=828)** | **Female Waist-Circumference** | | **P-value** |
| --- | --- | --- | --- | --- |
|  |  | **Low (N=350)** | **High (N=478)** |  |
| **Biochemical pregnancy** | 394 (47.6) | 178 (50.9) | 216 (45.2) | 0.107 |
| **Biochemical pregnancy loss** | 48 (5.8) | 19 (5.4) | 29 (6.1) | 0.698 |
| **Clinical pregnancy** | 346 (41.8) | 159 (45.4) | 187 (39.1) | 0.069 |
| **Multiple pregnancy** | 21 (2.5) | 9 (2.6) | 12 (2.5) | 0.956 |
| **Miscarriage** | 58 (7.0) | 20 (5.7) | 38 (8.0) | 0.213 |
| **Ectopic pregnancy** | 7 (0.9) | 3 (0.9) | 4 (0.8) | 1.000 |
| **Stillbirth** | 1 (0.1) | 1 (0.3) | 0 | 0.423 |
| **Live birth** | 280 (33.8) | 135 (38.6) | 145 (30.3) | 0.013 |
| **Twins** | 11 (1.3) | 3 (0.9) | 8 (1.7) | 0.372 |
| **Cumulative live birth** | 418 (50.5) | 198 (56.6) | 220 (46.0) | 0.003 |

**Table S4. Multivariable analysis for live birth and cumulative live birth**

|  | **Univariate regression** | | | | **Multiple regression#** | | | | | |
| --- | --- | --- | --- | --- | --- | --- | --- | --- | --- | --- |
|  | **cOR** | **95% CI** | | **P value** | **aOR** | **95% CI** | | | **P value** | |
| **Live birth** |  |  |  |  |  |  | |  | |  |
| **Female Waist-Circumference High vs Low** | 0.693 | 0.519 | 0.927 | 0.014 | 0.635 | 0.445 | 0.907 | | 0.013 | |
| **Cumulative Live birth** |  |  |  |  |  |  |  | |  | |
| **Female Waist-Circumference High vs Low** | 0.650 | 0.492 | 0.858 | 0.002 | 0.592 | 0.422 | 0.831 | | 0.003 | |

cOR: crude odds ratio,95%CI: 95% confidence interval, aOR: adjusted odds ratio.

**# Regression Model** for live birth and miscarriage was adjusted for female age, Female education level, female BMI, Type of embryo transfer, endometrial thickness, type of infertility, factors of infertility, number of IVF cycle, stimulation protocol, fertilisation method, stage of embryo transferred, number of embryo(s) transferred, male Waist-hip ratio.

**Regression Model** for cumulative live birth was adjusted for female age, Female education level, female BMI, type of infertility, factors of infertility, number of IVF cycle, stimulation protocol, fertilisation method, male Waist-hip ratio.

**Table S5. Odds ratios of live birth and cumulative live birth based on the joint multivariable regression analysis**

| **Female Waist-Circumference** | **Female BMI** | **N** | **Live birth** | | | | **Cumulative Live birth** | | | |
| --- | --- | --- | --- | --- | --- | --- | --- | --- | --- | --- |
|  |  |  | **aOR** | **95% CI** | | **P value** | **aOR** | **95% CI** | | **P value** |
| Low | Normal weight (18.5-<24) | 302 | ref | - | - | - | - | - | - | - |
|  | Overweight (>24) | 13 | 0.999 | 0.307 | 3.248 | 0.999 | 2.428 | 0.684 | 8.622 | 0.170 |
| High | Normal weight (18.5-<24) | 242 | 0.636 | 0.437 | 0.924 | 0.018 | 0.642 | 0.451 | 0.915 | 0.014 |
|  | Overweight (>24) | 235 | 0.773 | 0.530 | 1.127 | 0.180 | 0.706 | 0.492 | 1.013 | 0.059 |

aOR: adjusted odds ratio; 95%CI: 95% confidence interval.

**# Regression Model** for live birth and miscarriage was adjusted for female age, Female education level, Type of embryo transfer, endometrial thickness, type of infertility, factors of infertility, number of IVF cycle, stimulation protocol, fertilisation method, stage of embryo transferred, number of embryo(s) transferred, male Waist-hip ratio.

**Regression Model** for cumulative live birth was adjusted for female age, Female education level, type of infertility, factors of infertility, number of IVF cycle, stimulation protocol, fertilisation method, male Waist-hip ratio.

**Table S6. Neonatal outcomes of singletons in different waist-hip ratio groups**

|  | **N (%)** | **All neonates(N=269)** | | | **Males(N=142)** | | | **Females (N=127)** | | |
| --- | --- | --- | --- | --- | --- | --- | --- | --- | --- | --- |
|  |  | **Low**  **(N=93)** | **High**  **(N=176)** | **P value** | **Low**  **(N=50)** | **High**  **(N=92)** | **P value** | **Low**  **(N=43)** | **High**  **(N= 84)** | **P value** |
| **Gestational age (weeks)** | 38.8±1.8 | 38.7±2.4 | 38.9±1.3 | 0.609 | 38.8±2.1 | 39.0±1.3 | 0.572 | 38.7±2.8 | 38.8±1.4 | 0.839 |
| **Cesarean section (%)** | 169 (62.8) | 57 (61.3) | 112 (63.6) | 0.705 | 31 (62.0) | 62 (67.4) | 0.519 | 26 (60.5) | 50 (59.5) | 0.918 |
| **Preterm birth (%)** | 16 (6.0) | 6 (6.5) | 10 (5.7) | 0.800 | 4 (8.0) | 4 (4.4) | 0.452 | 2 (4.7) | 6 (7.1) | 0.716 |
| **Birthweight (g)** | 3296.9±497.1 | 3249.8±552.3 | 3321.8±465.1 | 0.260 | 3339.3±503.1 | 3430.8±423.4 | 0.252 | 3145.8±593.6 | 3202.4±481.6 | 0.564 |
| **Macrosomia (%)** | 11 (4.1) | 1 (1.1) | 10 (5.7) | 0.104 | 1 (2.0) | 6 (6.5) | 0.421 | 0 | 4 (4.8) | 0.299 |
| **LGA (%)** | 36 (13.4) | 9 (9.7) | 27 (15.3) | 0.194 | 5 (10.0) | 14 (15.2) | 0.383 | 4 (9.3) | 13 (15.5) | 0.334 |
| **Low birthweight (%)** | 16 (6.0) | 6 (6.5) | 10 (5.7) | 0.800 | 3 (6.0) | 3 (3.3) | 0.665 | 3 (7.0) | 7 (8.3) | 1.000 |
| **SGA (%)** | 9 (3.4) | 2 (2.2) | 7 (4.0) | 0.723 | 0 | 2 (2.2) | 0.541 | 2 (4.7) | 5 (6.0) | 1.000 |
| **Birth defect (%)** | 5 (1.9) | 3 (3.2) | 2 (1.1) | 0.344 | 1 (2.0) | 1 (1.1) | 1.000 | 2 (4.7) | 1 (1.2) | 0.265 |
